# Distant Metastasis in Colorectal Cancer Patients—Do We Have New Predicting Clinicopathological and Molecular Biomarkers? A Comprehensive Review

**DOI:** 10.3390/ijms21155255

**Published:** 2020-07-24

**Authors:** Stanislav Filip, Veronika Vymetalkova, Jiri Petera, Ludmila Vodickova, Ondrej Kubecek, Stanislav John, Filip Cecka, Marketa Krupova, Monika Manethova, Klara Cervena, Pavel Vodicka

**Affiliations:** 1 Department of Oncology and Radiotherapy, Charles University, Faculty of Medicine in Hradec Kralove, Šimkova 870, 50001 Hradec Králové, Czech Republic; jiri.petera@fnhk.cz (J.P.); ondrej.kubecek@fnhk.cz (O.K.); stanislav.john@fnhk.cz (S.J.); 2 Department of Molecular Biology of Cancer, Institute of Experimental Medicine of the Czech Academy of Sciences, Videnska 1083, 14220 Prague, Czech Republic; veronika.vymetalkova@iem.cas.cz (V.V.); ludmila.vodickova@iem.cas.cz (L.V.); klara.cervena@iem.cas.cz (K.C.); 3 Biomedical Centre, Faculty of Medicine in Pilsen, Charles University, Alej Svobody 1655, 32300 Pilsen, Czech Republic; 4 Institute of Biology and Medical Genetics, First Faculty of Medicine, Charles University, Albertov 4, 12800 Prague, Czech Republic; 5 Department of Surgery, University Hospital in Hradec Kralove, Sokolská 581, 50005 Hradec Králové, Czech Republic; filip.cecka@fnhk.cz; 6 The Fingerland Department of Pathology, University Hospital in Hradec Kralove, Sokolská 581, 50005 Hradec Králové, Czech Republic; marketa.krupova@fnhk.cz (M.K.); monika.manethova@fnhk.cz (M.M.)

**Keywords:** colon cancer, predictive markers, biomarkers, liver metastasis, metastatic colorectal cancer

## Abstract

Colorectal cancer (CRC) remains a serious health problem worldwide. Approximately half of patients will develop distant metastasis after CRC resection, usually with very poor prognosis afterwards. Because patient performance after distant metastasis surgery remains very heterogeneous, ranging from death within 2 years to a long-term cure, there is a clinical need for a precise risk stratification of patients to aid pre- and post-operative decisions. Furthermore, around 20% of identified CRC cases are at IV stage disease, known as a metastatic CRC (mCRC). In this review, we overview possible molecular and clinicopathological biomarkers that may provide prognostic and predictive information for patients with distant metastasis. These may comprise sidedness of the tumor, molecular profile and epigenetic characteristics of the primary tumor and arising metastatic CRC, and early markers reflecting cancer cell resistance in mCRC and biomarkers identified from transcriptome. This review discusses current stage in employment of these biomarkers in clinical practice as well as summarizes current experience in identifying predictive biomarkers in mCRC treatment.

## 1. Introduction

Colorectal cancer (CRC) ranks as the third most frequent cancer and the second leading cause of cancer-related death in developed countries [1]. Surgery represents one of the most important relevant approaches for CRC patients, and the only curative option for patients with localized and locoregional tumors, as well as for those with resectable distant metastases.

The five-year survival rate for stages I–III CRC is up to 80%, while for stage IV CRC, which represents 20% of all cases, it is about 13% [2]. Liver metastasis develops in almost 60% of patients with stage IV CRC [3]. The lung is the second most common site. In any case, the tumor-node-metastasis (TNM) staging system has been accepted in everyday practice due to its prognostic capability and simplicity of N staging. The status of the lymph nodes is also an important factor in determining the application of adjuvant chemotherapy after surgical resection [4].

Tumor metastasis, a multistep process, comprises dissemination of the cancer cells from the primary tumor to lymph nodes or remote areas, their survival in the microenvironment as single cells or micrometastases, and colonization of the distant organ by adaption of the tumor cells to the tissue microenvironment and subsequent proliferation leading to macrometastatic outgrowth [5]. The liver is the most frequent target of metastatic spread owing to the fact that most of the intestinal mesenteric drainage enters the hepatic portal venous system [6]. Nearly half of patients undergoing surgical resection for their primary CRC will eventually develop liver metastases [7]. If not treated, liver metastases exhibit an unfavorable prognosis with median overall survival (OS) of 5–20 months [8]. Currently, the surgical resection of isolated liver metastases represents the only curative approach. However, despite modern surgical techniques and adjuvant systemic therapy, only 20% of patients with distant metastasis achieve long-term remission (which ranges from 25.8–31.4 months when standard chemotherapy is administered), while 60–70% of patients develop local or distant recurrence [9,10]. According to historical data, only 5–10% of patients with liver metastases were resectable before the introduction of novel diagnostic and therapeutic methods [8]. At present, the resectability rates have reached to 20–25%. The resection of isolated pulmonary metastases increases the survival rates to 40% across 5 years [11].

Particularly during the last decade, the clinical outcome for patients with stage IV mCRC has improved substantially due to a more strategic approach to the delivery of systemic therapy [12]. Predictive biomarkers of chemotherapeutic efficacy are therefore required for choice of optimal mCRC treatment [13]. In the case of *RAS* wild-type tumors, a cytotoxic doublet with an anti-epidermal growth factor receptor (EGFR) antibody (cetuximab or panitumumab) seems to provide best results, but the combination of FOLFOXIRI (FOL–folic acid, FO–fluorouracil, OX–oxaliplatin, IRI–irinotecan) and bevacizumab (Avastin) remains the option [14]. On the other hand, for *RAS*-mutated diseases, the combination of bevacizumab plus either a cytotoxic doublet (FOLFOX or FOLFIRI) or FOLFOXIRI can be used [15,16]. However, by considering recent findings that right-sided mCRC may not respond well to anti-EGFR therapies, this paradigm has been questioned [17]. Furthermore, there is no consensus on which patients with up-front resectable disease and high-risk features should be offered neoadjuvant therapy. It is a common clinical practice to use adjuvant chemotherapy after surgical resection of distant metastasis, although there is still lack of evidence to support this approach in contrast to the situation in primary CRC; moreover, it is not clear which patients draw benefit from adjuvant therapy. Considering the extensive molecular and clinical heterogeneity between CRC and distant metastasis, it becomes apparent that an individualized approach based on molecular profiling will be necessary to achieve more promising results [18]. Currently, only clinical risk scores systems using standard pathological and clinical variables are at hand to stratify the patients with resected distant metastasis, as suggested by several authors in earlier studies [19,20,21]. The Fong clinical risk score (FCRS), a most well-known algorithm, assigns a single point for each of the following variables: A positive margin, extrahepatic disease, node-positive primary, disease-free interval from primary to metastases, number of hepatic tumors >1, largest hepatic tumor >5 cm, and carcinoembryonic antigen level >200 ng/mL. Based on this algorithm, patients are effectively stratified into those with a low risk, who demonstrated a 5-year OS of 47%, versus patients with a high risk, who demonstrated a 5-year OS of 24% [19].

However, clinical risk scores have limited impact. They came almost exclusively from a cohort from one institution reflecting patterns of local practice and bias and were not successfully verified across different institutions [22,23] in patients with longer follow-up or in neo-adjuvant chemotherapy settings [24]. Furthermore, none of these scores has achieved a level of prognostic reliability sufficient to influence clinical decision-making [25,26].

Besides, existing histopathological and molecular classifications are also insufficient for distant metastasis prediction that limits treatment strategy. Identification of cancer-related biomarkers can facilitate early diagnosis, patient outcomes, and recurrence risk [27].

About one third of patients undergoing primary tumor surgery relapse at distant sites [28]. Although certain histological factors, such as tumor differentiation, grade, and lymphovascular invasion, have been identified as higher risk features, there is still a lack of understanding of the molecular factors that may affect the disease recurrence [29]. Extensive CRC research over the last decade has suggested several molecular biomarkers, both of prognostic and predictive value. Although plenty of biomarkers have been extensively analyzed, very few of them were confirmed to be valid for management of CRC including defects in DNA mismatch repair (MSI phenotype), and *KRAS* and *BRAF* mutations [30]. Since the liver represents the most frequent site of metastatic dissemination in CRC (30–50%), the majority of studies investigating molecular prognostic and predictive markers in patients with mCRC included patients with liver metastasis, simply due to inadequate numbers of patients with metastases localized in other organs, such as lungs, accounting for 3% [31].

At the time of diagnosis, malignant tumors contain multiple cell populations with diverse biological heterogeneity that is not restricted to primary lesions [32,33]. The clonal origin and genetic heterogeneity of CRCs and heterogeneity between primary tumors and liver metastases have been recently addressed. It seems that the majority of CRC tumors is of polyclonal origin [34] and that half of liver metastasis is genetically distinct from their primary carcinomas [35,36]. Generally, metastases are now believed to carry identical mutations to the primary cancers from which they arise; however, other mutations occur during and after dissemination. Observed accelerated incidence of mutations in metastasis might subsequently result in genetic heterogeneity between primary and metastatic cancers [37,38].

## 2. Tumor Heterogeneity in Colorectal Cancer

CRCs exhibit significant level of heterogeneity. The inter-tumor heterogeneity (also known as interlesional heterogeneity) deals with differences between primary tumors appearing synchronously in the same patient, or between a primary tumor and its corresponding metastases. On the other hand, intra-tumor heterogeneity (also known as intralesional heterogeneity) indicates the differences within the tumor. Intra-tumor heterogeneity also means the presence of different morphological, inflammatory, genetic, or transcriptomic subclones in a single tumor, which affects the outcome of the disease and the therapeutic response. Heterogeneity can also be divided as spatial or temporal. Spatial heterogeneity characterizes variations in different tumor regions, i.e., either different genetic subpopulations within the primary tumor or differences between the primary tumor and its metastatic lesions [39], while temporal heterogeneity assigns to changes developed over time in the individual tumor [33].

The investigation of tumor heterogeneity is of great clinical interest because it affects treatment decisions. Tumors consist of a mosaic of cancer cells with distinct features and different sensitivities to anti-cancer therapies. Natural selection results in clonal expansion, leading to different subclones with different abilities for proliferation, migration, and invasion [40]. In CRC, tumor heterogeneity was associated with worse prognosis and patient outcomes [30,32]. Intra-tumor heterogeneity was also considered as one of the major reasons contributing to chemoresistance and treatment failure, and one of the dominant causes of worse survival in patients with distant metastases [41].

Several studies focusing on the study of tumor heterogeneity are restricted to the comparison of primary tumors and adjacent non-malignant tissues; however, detailed research on corresponding metastases is needed [42,43]. Thanks to the rapid development of new omics technologies, the current research has also focused on comparing the difference between the primary tumor and distant metastases. Several studies have reported high genomic concordance between primary CRC and liver metastases [44,45]. The presence of lymph node metastases in CRC patients may be considered as a prognostic factor. Besides, lymph node metastases are thought to be the precursors of distant metastases and their surgical resection is necessary to achieve a “cancer-free” state [46]. Cady [47] proposed an alternative model that distant metastases occur independently of lymph node metastases. Recently, Naxerova et al. [48] observed that the majority of distant metastases and those in lymph nodes derived from independent subclones in the primary tumor, whereas in one third of cases they shared common sub-clonal origin. These results pointed to two different relationships between lymphatic and distant metastases.

Comprehensive gene expression profiling of the primary CRC and matched metastases may identify the molecular events involved in tumor progression [49]. Lee et al. [50] showed a high agreement of gene expression signature between the primary tumor and liver metastases. However, the fusion transcripts were expressed differently between primary CRC and liver metastases. Similarly, Vermaat et al. [51] noticed a considerable loss or gain of genetic variants between primary tumors and corresponding metastases. It can be hypothesized that genetic analysis of metastases represents predictive value for selection of patients for specific treatment regimes.

Recently, liquid biopsy approach has gained a leading position in cancer research, and one of the advantages that makes it particularly promising is its potential to overcome the problem of tumor heterogeneity [52,53,54]. The tissue biopsy from the specific part of tumor may not represent the genetic profile of the tumor. Liquid biopsy is more representative of the entire tumor and enables real-time monitoring of tumor progression.

## 3. Sidedness of the Primary Tumor

Tumors originating from the left side of the colon harbor distinct molecular properties, biological behavior, and prognosis as compared to those arising from the right side [38]. Furthermore, tumor sidedness is now generally accepted as one of the factors guiding a treatment choice, as there are differences regarding the tumor response to targeted therapies. The patients with left-sided tumors tend to have liver-only metastases [55]. These patients have lower tumor burden in terms of the number of metastases and the number of segments involved, higher resectability rate, and markedly better survival than those with right-sided tumors [56]. Right-sided tumors are more often poorly differentiated and *KRAS-* and/or *BRAF*-mutated [57]. *RAS* mutations occur in 35–45% of all patients with mCRC [58], while in the population with resectable liver metastases, it is 10–15% lower, indicating the negative prognostic value of a *RAS* mutational status and its association with the likelihood of surgical resection [59]. There is an emerging question regarding whether clonal selection during the metastasizing process leads to a shift in molecular patterns, and to which extent liver metastases retain their original molecular profile.

## 4. The Cancer Stem Cell Model

Recently, the cancer stem cell (CSC) model has been proposed to explain tumor heterogeneity. The detection of stem cells in solid tumors has also highlighted their important role tumorigenesis. Stem cells, often called CSCs, comprising a small minority of neoplastic cells within a tumor, have been experimentally characterized for the capacity to seed new tumors and create populations other than CSCs without the tumorigenic potential. CSC subpopulations were observed in a number of malignancies [60] and CSC-rich tumors were associated with aggressive disease and worse prognosis [61].

**Figure 1:**
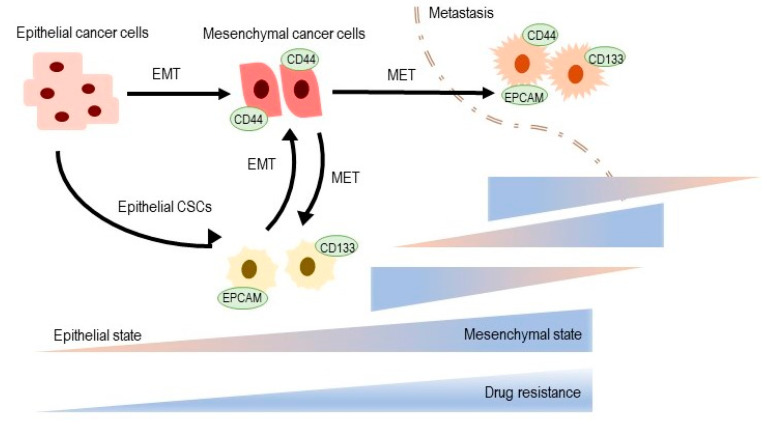
The epithelial–mesenchymal transformation (EMT) and cancer stem cells (CSC) at the crossroads towards drug resistance [68,69]. CSC subpopulations show EMT phenotypes. See text for details. MET: Mesenchymal-epithelial transformation.

Epithelial to mesenchymal transition (EMT) is a reversible process important for tissue and organ formation, through which non-motile epithelial cells with tight cell adhesions and apical basal polarity are converted to individual motile mesenchymal cells with a front to promote polarity [62]. When a tumor metastasis arises, often by EMT [63], disseminated cancer cells require a self-renewal ability similar to that of CSC to form macroscopic metastases (Figure 1). This increases the possibility that the EMT process, which allows cancer cells to spread, may also confer the ability of self-renewal to spread cancer cells [64]. The EMT process thus connects clonal evolution and CSC models and creates the basis for a phenotypic model of plasticity [65]. In many cancers, only neoplastic cells in the CSC-enriched subpopulation show forms of EMT activation [64,66,67].

Drug resistance may inhere in cancer stem-cells (CSCs) populations that have been detected in a variety of tumors, including CRC. The CSCs, also known as tumor-initiating cells (TICs), are believed to play a role in chemoresistance and cancer relapse [70]. They are involved in tumor growth and metastatic spread, but most tumors consist of non-tumorigenic cells that are not capable of metastatic seeding or tumor progression [33]. The CSCs share several normal stem cells features that provide a long existence, including the relative silence, dormancy, resistance to chemotherapeutic agents and toxins through the expression of drug efflux transporters, an active DNA-repair capacity and a resistance to apoptosis, vascular niche, stability to hypoxia, and enhanced activity of repair enzymes [71]. Understanding the characteristics of CSCs is crucial to lay the foundation for new generation in treatment of cancer and their identifying and eliminating represents a significant help to diminish drug resistance [72].

These tumor-initiating cells have been reported in the literature through specific surface proteins, such as CD133, CD166, or CD44 [73,74], which on average account for 11.4% of epithelial cells in primary CRC [75]. CD133 is one of the most commonly used markers to identify CSCs, but its specificity is controversial. Recent studies have shown that the AC133-CD133 epitome is responsible for the identification of CSCs [76]. The expression of CD133 or co-expression of CD166/CD44 or CD24/CD44 cannot sufficiently identify CSCs populations. This has raised some doubts as to the suitability of using CRC cell lines for screening CSC-specific therapies [77].

Rocco et al. have found that although CD133(+) and CD133(+)/CD44(+) expressions were detectable in human primary gastric cancers (GCs), they either did not explicit stem cell properties or demonstrate tumor-initiating properties in xenograft transplantation experiments [78]. Based on meta-analysis investigating the CD133 role in CSCs, CRC patients with CD133Low expression receiving adjuvant therapy exhibited longer survival than those with CD133High. These findings suggested that CD133 could serve as a predictive marker of poor prognosis and treatment failure in CRC [79]. In addition, CD133 and CD44 were simultaneously expressed in patients with liver metastasis [80]. Epithelial to mesenchymal transition (EMT) was involved in CSCs where HCT116 p53+/+ colon cancer cells with a high expression of CD133/CD44 showed EMT after long-term culture [81]. Similarly, Dylla et al. demonstrated that the CSCs fraction is increased in CRC tumors after chemotherapy and may help explain relapse following treatment [82]. In another clinical study, the CD133 CRC stem cell marker was followed. Three hundred and three patients with CRC stage I to III who underwent surgical resection were found to have higher CD133 expression that correlated with poor prognosis after radical resection [83]. The prognostic value of co-expression of two CSCs biomarkers, CD44 and CD133, with wild-type EGFR (wtEGFR) and EGFRvIII in CRC patients, was studied and CD133/CD44 expression was associated with primary resistance to irinotecan and acquired resistance to anti-EGFR inhibitors in vitro. The results suggest that co-expression of these markers and EGFRvIII may be potential biomarkers of poorer overall survival and resistance to therapy in patients with mCRC [84].

Based on results from a recent meta-analysis that analyzed the correlation of expression of surface markers and CSCs (tumorigenicity) properties by monitoring tumor incidence and volume in vivo, authors suggested that CD133 expression may represent a strong biomarker for identifying of CSCs in primary tumors and can be designed as a prognostic marker in CRC patients [85,86].

## 5. Distant Metastasis-Related Biomarkers in Clinical Practice

There were attempts to use clinical risk scores to stratify patients according to the risk of recurrence after metastasectomy, however, with only limited success. The first prognostic gene expression signature for OS after resected distant metastasis that was also externally validated showed dominance over clinical risk scores and highlighted the prognostic potential of transcription profiling [55,59]. Similarly, there is a need to identify biomarkers predictive of tumor response to targeted agents. Currently, the only validated and clinically used predictors of responses to anti-EGFR therapy are represented by *KRAS, NRAS*, and *BRAF* mutational status. A good concordance (almost to 95%) in the presence of mutations in high risk genes, such as *KRAS, TP53, APC, PIK3CA, BRAF,* and *NRAS*, between the primary tumor and its metastases have been reported [87,88]. However, tumor heterogeneity in mCRC have been identified. Intra-tumoral and inter-tumoral heterogeneity of *KRAS* mutations was observed in mCRC and corresponded with resistance or lower efficacy of anti-EGFR therapies [89]. For instance, Jeantet et al. determined a high amount of heterogeneity in distribution of *RAS* mutations in mCRCs; in detail, 33% of intra-tumoral and 36% of inter-tumoral heterogeneity [90]. Nevertheless, in light of recent findings that right-sided *RAS* and *RAF* wild-type tumors do not respond well to anti-EGFR therapies [55], there is an emerging question regarding whether anti-EGFR therapy should be given only to liver metastasis originating from left-sided CRC. There is growing evidence that other biomarkers, including mutations of *PI3KCA, FBXW7, SMAD4*, and others, may evince predictive value. However, further validation is needed. It is becoming strikingly clear that multigene expression assays might help to select the most effective induction therapy as well as identify a high-risk population suitable for adjuvant therapy. It should be kept in mind, however, that multigene expression assays are sensitive to sample quality and pre-analytical handling [91]. Moreover, gene expression may not reflect the final function of the protein. Similarly, the tumor microenvironment and immune system play a major role in tumor progression and response to therapy. The presence of specific immune cells within the tumor tissue, such as increased densities of T cell infiltrates with a high proportion of CD8+ T and demonstrated that immune reactivity at the tumor site influences clinical outcome [92]. Primary colorectal carcinomas were associated with a significant protection against tumor recurrence and prognosis was especially marked in deficient mismatch repair system (dMMR/MSI) tumors [93]. In detail, hypermutated dMMR/MSI of mCRCs evince higher sensitivity to inhibitors of immune checkpoint that revive cytotoxic T cells to eliminate dMMR/MSI tumors cells [94]. Patients with dMMR/MSI tumors greatly benefit from immunotherapy, regardless of the tumor type, with disease control rates of 80% and OS superior to three years in chemo-resistant mCRC [95]. Consequently, all mCRCs should be regularly tested for MMR and MSI status [96].

In mCRC, the dMMR/MSI phenotype (around 5% of mCRCs) is associated with worse prognosis and chemoresistance [95,97]. However, recent studies have reported prolonged OS in dMMR/MSI mCRC after anti-vascular endothelial growth factor (anti-VEGF) treatment, as compared with anti-EGFR, however without change in survival conferring to chemotherapy regimen, i.e., irinotecan-based chemotherapy, in contrast to oxaliplatin-based chemotherapy [98]. Finally, recent nonrandomized trials point to the high efficacy of the immune checkpoint inhibitor in dMMR/MSI chemo-resistant mCRC as a reason for the high tumor mutational burden. Another response predictor to the immune checkpoint inhibitor is the immunohistochemistry labeling of the programmed death-ligand 1 (PD-L1) protein [96,99].

Both immunological and molecular markers seem to provide promising prognostic and predictive information in distant metastasis, but this topic warrants further research [93,94,98].

The management of mCRC has been markedly changing in recent years with the introduction of targeted therapies resulting in significant improvements in survival and quality of life of patients. Currently, the gold standard of systemic therapy in the first or second line is a combination of chemotherapy and targeted therapy. The combined chemotherapy regimens are based on 5-fluorouracil (5-FU) and leucovorin with oxaliplatin (FOLFOX) or irinotecan (FOLFIRI), or both (FOLFOXIRI) [72]. The monoclonal antibody (mAb) against vascular endothelial growth factor (VEGF) bevacizumab, VEGF-trap aflibercept and mAbs against epidermal growth factor receptor (EGFR) cetuximab and panitumumab represent the widely used targeted agents combined with the first- or second-line chemotherapy [100].

Anti-EGFR mAbs are used for patients with tumors harboring the wild-type *RAS* gene, which represents a well-established predictive biomarker. The efficacy of anti-EGFR mAbs was restricted to patients with tumors harboring the wild-type *KRAS* gene. Thus, *KRAS* gene mutations, occurring in 35–45% of cases, became the most important predictive biomarker in mCRC patients [101,102,103,104]. Extended RAS analyses have demonstrated a lack of response to anti-EGFR mAbs also in patients with tumors harboring *NRAS* gene mutations, in 1–6% of cases [105,106,107].

It was also assumed that the *BRAF* oncogene evinces more prognostic significance [108]. *BRAF* mutations can be used as an effective predictive biomarker for BRAF-targeted therapies. The combination of encorafenib (BRAF inhibitor), binimetinib (MEK inhibitor) and cetuximab in mCRC patients with tumors harboring *BRAF* mutations resulted in significantly longer overall survival and a higher response rate to therapy [109]. mCRC microsatellite instability (MSI) phenotype in mCRC is associated with worse prognosis and chemoresistance to standard treatment [94]. On the other hand, the MSI-high tumors often respond to immunotherapy, and two programmed cell death 1 (PD1)-blocking mAbs, pembrolizumab and nivolumab, have been effective in mCRC patients with MSI-H tumors.

Considerable efforts are currently dedicated to identifying biomarkers associated with therapy response. The identification of these biomarkers is crucial for individualized treatment strategies in mCRC patients.

Despite *RAS* gene mutations representing effective and currently the most important predictive biomarkers, there is still a proportion of patients with tumors harboring the wild-type *RAS* gene, who derive no or poor benefit from the systemic therapy containing anti-EGFR mAbs. Moreover, for the antiangiogenic targeted therapy represented by bevacizumab and aflibercept, there are no predictive biomarkers available in the routine clinical practice. *BRAF* mutations and MSI-H represent other recently established predictive biomarkers for novel therapies. However, they are relatively rare, occurring in approximately 5–10% and 3–5% of mCRC, respectively [94].

There is an urgent need for personalized medicine to select the optimal therapy from an expanding range of the systemic treatment modalities in mCRC patients. The identification of biomarkers that could predict the response to a specific type of systemic therapy and/or be used in the effective noninvasive monitoring of mCRC patients treated with palliative systemic therapy might the shift in the therapy towards a precision medicine.

## 6. Side Effects and Toxicity

The therapeutic CRC options have increased during the last 20 years, and the complexity of decision-making has also advanced. The treatment strategies in neoadjuvant, adjuvant, and palliative approaches differ, and treatment decisions influence not only the drug efficacy. Age, the presence of significant comorbidities, and various treatment regimens and strategies provide medical oncologists with a number of options to try to maximize patients’ quality of life and longevity.

Systemic chemotherapy plays a major role in the CRC management. However, cancer treatment can be significantly prolonged and obstructed due to the presence of chemotherapy-induced side effects which may require dose reduction or delay or discontinuation of treatment. Although 5-FU is one of the safest chemotherapeutics, several CRC patients still have serious side and toxic effects. Clinical manifestations of 5-FU toxicity include fever, fatigue, mucositis, stomatitis, nausea, vomiting, and diarrhea [110]. Other common toxic effects include leukopenia, neutropenia, thrombocytopenia, anemia, neuropathy, skin rash, and hand–foot syndrome [111]. Neurological abnormalities, such as cerebellar ataxia and changes in cognitive function, have also been reported rarely and have occurred in less than one percent of patients [112]. The oxaliplatin side effects includes peripheral neuropathy, nausea and vomiting, diarrhea and fatigue [113,114]. However, most of the patients do not experience any of these side effects.

Although it has been reported that the patient outcomes have improved after the addition of oxaliplatin or irinotecan to the 5-FU regimen, the toxicity has also increased [115]. The efficacy of 5-FU/LV combined with oxaliplatin (FOLFOX) or irinotecan (FOLFIRI) in the first-line treatment of mCRC is comparable [116]. The combination therapies FOLFOX and FOLFIRI have become established as efficacious cytotoxic regimens for the treatment of mCRC, resulting in overall improvement in survival of approximately 2 years [117].

## 7. Drug Resistance in mCRC

Drug resistance is the major cause of treatment failure in CRC. Resistance can be intrinsic (primary) or acquired (secondary). Both intrinsic resistance, characterized by cancer cells that have little or no response to treatment from the beginning, and acquired resistance, where the tumor may become cross-resistant to a range of chemotherapies, lead to treatment failure in over 90% of patients with mCRC [72].

Malignant tumors can possess intrinsic and/or acquired resistance and each is important in determining initial and subsequent lines of treatment. Innate resistance is typically noted during early drug development or in early phase clinical trials of biologic efficacy. For instance, resistance to EGFR antagonists was initially not well understood and in early studies only 10–20% of patients exhibited a response to the EGFR-targeted therapies, cetuximab or panitumumab [118]. The subsequent elucidation of *RAS* mutations in CRC clarified a marker of innate resistance to these therapies and changed their clinical use.

Different mechanisms of acquired resistance can exist for each cytotoxic therapy and each targeted pathway, but often acquired resistance to one drug confers resistance to other drugs which may work by different mechanisms of action, a concept referred to as multidrug resistance (MDR). In general, resistance to traditional cytotoxic therapy is accomplished by decreasing the delivery of drugs to the cancer cell, either by increased efflux out of the cell mediated by ATP-dependent transporters, by decreased uptake into the cell, or by a change in enzymes involved in metabolism. Alternately, resistance can be conferred by changes within the cell itself by genetic or epigenetic modifications that can alter drug sensitivity.

Albeit targeted drugs may provide more adequate therapy, studies have displayed moderate benefits and, like traditional chemotherapy, both primary and secondary resistance may arise [119].

Chemoresistance may be involved in the disease relapse and in the process of metastasis development. It restricts the improvement of clinical outcomes for cancer patients and persists as the main obstruction to cancer therapy. Therefore, it is very important to understand the molecular mechanisms of chemoresistance to find novel therapeutic approaches. Currently, it is believed that therapy resistance is the consequence of a clone selection during therapy and the presence/generation of novel mutations in cancer cells that enable them to resist the cytotoxic attack [120]. Kim et al. have shown that CRC seems to follow the clonal evolution concept [121]. In this model, the gradual gain of mutations or non-genetic alterations leads to the development of specific sub-clones with different abilities to adapt to the tumor microenvironment, resulting in inter- and intra-tumoral heterogeneity [33]. mCRC often had one clone that is dominant with several minor sub-clones, while a preeminent clone was found less frequently in early stage CRC. In distant metastases, *KRAS* and *TP53* mutation heterogeneity was rare. This reflection of decreasing tumor heterogeneity together with tumor progression, particularly in distant metastases, was promoted by study of Kim et al., who demonstrated a higher number of mutated alleles in CRC metastases compared to matched primary tumors, aiming at contraction in genetic and non-genetic heterogeneity in distant metastases [96,121].

On the other hand, Sottoriva et al. proposed a different aspect of heterogeneity leading to chemoresistance by highlighting the timing of tumor mutations. This “big bang model” considers that the mutations that are responsible for tumor formation and progression appear early in CRC. Therefore, the biological behavior of a tumor is set early and may clarify why some large tumors never form metastases, and other small tumors develop metastases earlier. Thus, in this model, mutations that lead to evolution of different tumor sub-clones could be considered as passenger mutations rather than induced by Darwinian option of the “fittest” sub-clone, which leads to spatial dominance [122].

Clearly, new and effective strategies to overcome chemoresistance are urgently needed. Malignant tumors may have an intrinsic resistance and/or acquired resistance, and both are important in determining initial and subsequent lines of treatment. Innate resistance of cancer cells is typically noted during an early drug application. But for instance, resistance to EGFR antagonists was initially not well understood and in early studies only 10–20% of patients exhibited a response to the EGFR-targeted therapies cetuximab or panitumumab [118]. The subsequent elucidation of the role played by *RAS* mutations in CRC clarified a marker of acquired resistance to these therapies and changed their clinical use [57].

## 8. Experience in Predicting Markers

Patients with distant metastasis show a wide heterogeneity of clinical outcomes. Many systems have been developed to determine patient prognosis based on individual tumors and personal characteristics to understand who would benefit most from hepatic resection. Such a strategy also aids in adapting an individualized treatment strategy that focuses on providing appropriate, timely, and personalized therapy. In addition, in the period of modern effective chemotherapy and multidisciplinary management, traditional tumor and patient characteristics may have less impact on survival. Recent research has focused on the use of genetic biomarkers and molecular signatures to identify subgroups of patients most likely to obtain benefits from a given therapy and to use better survival estimates to guide individualized patient treatment plans [123].

Gene expression profiling of cancer cells has been used for decade to accurately classify tumors and to identify markers predicting the patient’s outcome. In the case of primary CRC tumors, numerous expression studies have been conducted so far [124,125,126].

Plentiful studies analyzing either whole transcriptome or small sets of expressed genes reported significant differences in the gene expression profiles between non-metastatic and mCRC [127,128]. A few other studies also described variances in single gene expression between primary tumor and/or liver metastasis on one side, and lung metastasis on the other side [129,130]. More data are available from expression-profiling analyses of clinical samples and/or animal models with CRC liver metastasis. In these studies, various gene signatures for colon-to-liver metastasis were suggested [131,132]. Although many identified signatures or molecular markers have been validated by other groups, yet none of them has been used as a diagnostic or prognostic tool applicable to clinical practice as reviewed [133,134]. In order to obtain a real genetic signature for distant metastasis by transcription profiling, further studies are needed to improve reproducibility and increase consistency, and the validation of results needs to be implemented. Before the clinical use can be implemented, prospective studies should be performed with a huge number of patients to accomplish reproducible results. Improving of our knowledge of molecular pathways involved in the development of distant metastasis will lead to a better approach to the prevention and treatment of this disease.

Identification of appropriate biomarkers can significantly improve the choice of treatment strategies for patients with CRC. Most of these markers can notify the oncologists about the overall prognosis of the disease; nevertheless, they fail to make a therapeutic choice. In fact, the majority of identified biomarkers, with the exception of the *KRAS* and *BRAF* genes and MSI status, did not predict therapeutic responses or did not reach the clinical practice.

Recently, Koncina et al. [135] and Lee [136] outlined new clinicopathological and molecular biomarkers for all CRC that can be translated into the clinical setting; however, only few of them are applicable for mCRC.

Among them, the liquid biopsy approach showed the ability to provide the most clinically relevant information. To ensure the optimal clinical treatment of patients with mCRC, it is important to regularly evaluate the effectiveness of treatment. Monitoring of concentration changes of circulating tumor DNA (ctDNA) might provide an opportunity to personalize treatment. ctDNA may be helpful in determining the response to disease treatment in patients with mCRC: 1) As a predictive biomarker for treatment selection; and 2) as a monitoring tool for response to treatment [54]. Several studies have proposed the use of ctDNA for monitoring patients after surgery and identifying patients at high risk of recurrence (Table 1).

In general, the gene fusion ranges between 0.5–2% in patients with CRC, but reaches 4% in MSI high mCRC [175,176,177]. The prognostic and predictive value of several gene fusions is still not clear [178]. The U.S. Food and Drug Administration (FDA) approved the neurotrophic tyrosine receptor kinase (NTRK)-inhibitor entrectinib and larotrectinib in NTRK-fusion-mutated tumors of all organ types, including CRC, provided they do not contain a known acquired resistance mutation, in 2019.

HER2 (Errb2) is a transmembrane receptor of the EGFR family and its activation stimulates cell proliferation and the inhibition of apoptosis. Amplifications of HER2 occur in 2–6% of mCRC, but reach up to 13% in wild-type KRAS/NRAS/BRAF mCRC [179]. HER2 has recently gained much importance in CRC. Two recent clinical trials, MyPathway (NCT02091141) and HERACLES (NCT03225937), have shown encouraging clinical benefits for a dual HER2 blockade in mCRC patients with HER2 amplification.

The CRC classification by consensus molecular subtypes of CRC (CMS) proposed by Guinney et al. [165] subtypes might serve as a prognostic biomarker. These 4 subtypes are: CMS, with MSI and immune activation (14%); CMS2, with canonical CRC alterations (37%); CMS3, with metabolic dysregulation (13%); and CMS4, with mesenchymal features (23%). However, from a clinical point of view, CMS does not seem to have superior value to routinely use clinical indication criteria for selecting patients for optimal treatment with either anti-EGFR or anti-VEGF agents. Overall, CMS categorization provides a detailed insight into CRC etiology but currently still has no real impact on clinical decision-making. However, the inclusion of patients with distinct CRC molecular subtypes represents an essential start for clinical translation. The prognostic significance of CMS subtypes in both early and metastatic CRCs bolsters the fact that they could be used in the assessment of therapy responses and might aid the treatment choice.

The immunoscore is a scoring system that is based on the quantification of cytotoxic and memory T cells in the core of the tumor and in the tumor’s invasive margin [172,180,181,182]. It represents a strong prognostic marker in CRC [170,183,184,185] and has a dual advantage over TNM staging. The immunoscore has been reported to be a better predictor than MSI alone [174]. Due to the important role of host immunity in controlling tumor progression, it is probably necessary to include the immunoscore in the cancer classification.

As stated before, the choice of the treatment strategy depends on various clinical factors and biomarkers. Several other molecular biomarkers have been also suggested, such as miRNAs, the CpG island methylator phenotype (CIMP). Recently, the expression of miR-31-3p was introduced as a promising predictive biomarker for anti-EGFR therapy in patients with the wild-type *KRAS* gene, and treated with adjuvant chemotherapy. Low miR-31-3p expression in patients treated with standard chemotherapy and cetuximab was associated with longer progression-free survival compared to patients expressing high levels of miR-31-3p [186,187,188,189,190]. The CIMP-positive tumors (hypermethylation of at least three out of five pre-defined marker) displayed independent biomarker or recurrence in CRC [191,192,193,194]. Further studies are urgently needed to identify and validate new biomarkers to improve outcomes in patients with CRC.

## 9. Conclusions and Future Perspectives

The metastatic process into specific organs is largely dependent on the ability of a tumor cell to interact with its microenvironment that is influenced also by its internal characteristics [195]. The identification of these cell characteristics is of prime interest, as it could lead to the design of new therapeutic strategies. CRC research is aimed at several areas, such as prevention, early stage cancer identification, prognostic and predictive markers recognition, new molecular targets identification, drug development, and clinical practice adjustment. Identification of major cancer pathways on genetic, proteomic, and epigenetic level contributes to our better understanding of CRC behavior. Recent years witnessed substantial progress in understanding of microenvironment/microbial settlement in CRC onset, pathogenesis, and progression. The future precise risk stratification of patients with advanced CRC and subsequent pre- and post-operative decisions must not omit consideration of microenvironmental (microbial) context (e.g., based on liquid biopsy).

Despite the improving knowledge of the development and progression of CRC, results in common clinical practice treatment show only little advance.

Starting in 2016, relevant sub-analysis of 6 important clinical trials (FIRE-3, CALGB/SWOG-80405, PEAK, Clinical Study No 20050181, CRYSTAL and PRIME) [196] opened the question of primary tumor sidedness as an additional character for molecular tumor profile and dual inhibition (VEGF/EGFR) treatment efficacy [197]. These findings resulted afterwards in standard use of consensus molecular subtypes (CMS) in the majority of newly designed trials [165,175]. The findings in this area would help us to identify, for example, patients at higher risk of tumor recurrence after surgery and treatment or development of distant metastasis. Moreover, small subpopulations of patients with druggable molecular changes are already treated correspondingly, for instance *BRAF*-mutated tumors (10%), HER2-positive tumors (3%), NTRK -fusion positive tumors (1–2%), FGFR (fibroblast growth factor receptor)-amplified tumors or ROS1 (receptor tyrosin kinase)- and ALK (anaplastic lymphoma kinase)-fusion-positive tumors (up to 1%) [175,198,199,200]. Most expectations are put into immunomodulation with PD-1 or PD-L1 antibodies, currently used in MSI/MMR-deficient tumors [201]. Great efforts have been undertaken to change the immune cold tumor environment into the hot one and to take the advantage of natural property of the immune system to precisely and accurately target specific antigens [202]. Another interesting approach to treatment is the use of epigenetic modification. Notably, HDACi (histone deacetylase inhibitors), namely Vorinostat, are already used in hemato-oncology; however, the results are still waiting for application in the CRC treatment [203]. Besides the detection of specific DNA mutations for predicting responses to anti-EGFR therapies [149], the liquid biopsy approach as measuring the plasma/serum concentrations of cell-free DNA (cfDNA) and/or circulating tumor DNA (ctDNA) emerged to be an effective indirect predictive biomarker in mCRC patients [53,204].

Outcomes after surgery for distant metastasis still remain highly heterogeneous, ranging from death within 2 years to a long-term cure. The further improvement of the prognosis highlights the need for an implementation of a spectrum of criteria (listed in Table 2, A–F) to assist the stratification of patients with advanced CRC to ensure optimal pre- and post-operative cure.

All new findings will help us further adapt the known CRC treatment. Although these efforts may take a long time, new and exciting discoveries seem to be closer than ever.

## Figures and Tables

**Table 1:** The list of biomarkers used in clinical practice of metastatic colorectal cancer (mCRC).

Biomarkers	Clinical Relevance	Therapy Benefit	Without Benefit on Therapy	References
Tumor sidedness	Predictive Prognostic	FOLFIRI plus cetuximab in patients with left-sided CRC	Right-sided CRC associated with worse outcomes	[55,137,138,139]
		Immunotherapy in right-sided CRC	Anti-EGFR therapy for right-sided CRC	
ctDNA	Predictive Prognostic	Targeted therapy	-	[140,141,142,143,144,145,146,147,148,149,150,151,152,153,154]
		Systemic chemotherapy		
Tumor burden	Predictive Prognostic	Immunotherapy	-	[155,156,157]
ALK, ROS1, NTRK1-3 fusions	Predictive	ALK, ROS and TRK inhibitors	-	[158,159,160]
HER2 amplification	Predictive	-	Anti-EGFR therapy	[161,162,163,164]
The consensus molecular subtypes of colorectal cancer (CMS)	Prognostic	-	-	[165,166,167,168,169]
Immunoscore	Prognostic	-	-	[170,171,172,173,174]

**Table 2:** Perspectives for colorectal cancer.

Perspectives	Factors
A. Cancer prevention	Lifestyle risks
	Hereditary cancer identification and management
	Public education
B. Identification of early CRC stages	Screening programs (population coverage)
	Improvement of current tests
	New low-invasive methods (liquid biopsies)
C. New prognostic and predictive markers necessary to distinguish suitable patients for current treatment	Patients suitable for neoadjuvant/adjuvant chemotherapy
	High-risk patients where more aggressive approach is applicable
	Predictive markers for targeted therapy
D. Identification of new molecular targets	Anti-tumor immunity activation
	Epigenetic changes
	Role of microbiome
E. Drug development	New effective molecules against identified targets
	Treatment for larger groups of patients (role of immunotherapy)
	Reduced toxicity and side effects of the treatment
F. Faster implication of actual knowledge to the clinical practice	Clinical trials design improvement
	Cost efficiency vs. clinical efficacy acceptable equilibrium
	Suspension of treatment, which has not shown efficacy
